# Female Sex Is Independently Associated With Greater Sagittal Tibial Tubercle‐Trochlear Groove Distance and Patellar Tilt in High‐Grade Trochlear Dysplasia

**DOI:** 10.1002/ars2.70161

**Published:** 2026-11-09

**Authors:** Martin Hartmann, Berfin Caliskan, Jakob Ackermann, Sandro F. Fucentese

**Affiliations:** ^1^ Department of Orthopedics and Trauma Surgery Lucerne Cantonal Hospital Luzern Switzerland; ^2^ Department of Orthopedics Balgrist University Hospital, University of Zurich Zurich Switzerland; ^3^ Rush University Medical Center Chicago Illinois U.S.A.

## Abstract

**Purpose:**

To assess sex‐related differences in patellofemoral (PF) anatomy among patients with PF instability and high‐grade trochlear dysplasia (Dejour types B‐D).

**Methods:**

Consecutive patients with symptomatic PF instability and high‐grade trochlear dysplasia (Dejour types B‐D) who were scheduled for surgical treatment between January 2010 and December 2020 and had available preoperative radiographs and magnetic resonance imaging were retrospectively identified at a single center. Patients with normal trochlear morphology, type A dysplasia, missing preoperative magnetic resonance imaging, or prior osseous procedures were excluded. Standardized magnetic resonance imaging‐based measurements were obtained for trochlear morphology, tibial tubercle position, and patellar geometry. Depending on data distribution, parametric or nonparametric tests were used to compare sexes. Multivariate linear mixed‐effects models were performed to calculate regression coefficients (*B*) with 95% confidence intervals, adjusting for patient clustering, age, body height, supratrochlear spur height, Caton‐Deschamps index, sulcus depth, and tibiofemoral rotation.

**Results:**

A total of 301 knees from 278 patients were analyzed; 192 knees (63.8%) were from female patients. The mean age was 22.6 ± 8.1 years (range, 11‐50 years), and the mean body mass index was 24.9 ± 5.5 (range, 16.4‐48.1). Distribution according to the Dejour classification was 40.5% type B, 31.2% type C, and 28.2% type D. Female patients showed smaller patellar width (37.9 ± 3.4 mm vs 41.3 ± 4.1 mm; *P* < .001) and thickness (16.8 ± 2.0 mm vs 18.5 ± 2.6 mm; *P* < .001), as well as greater patellar tilt (29.3° ± 10.7° vs 25.0° ± 10.8°; *P* = .001) and sagittal tibial tubercle‐trochlear groove (sTTTG) distance (9.9 ± 5.2 mm vs 8.4 ± 6.0 mm; *P* = .005). After adjustment for age and body height, differences in patellar width, patellar thickness, patellar tilt, and sTTTG remained significant. After additional adjustment for supratrochlear spur height, Caton‐Deschamps index, sulcus depth, and tibiofemoral rotation, patellar tilt (*B* = 3.51°, *P* = .021) and sTTTG distance (*B* = 2.36 mm, *P* = .004) remained greater in female patients.

**Conclusions:**

Among patients with PF instability and high‐grade trochlear dysplasia, female sex was associated with greater patellar tilt and sTTTG distance after adjustment for age, body height, and relevant anatomical covariates.

**Level of Evidence:**

Level III, prognostic retrospective comparative study.

Trochlear dysplasia (TD) is a well‐established anatomical risk factor for patellofemoral (PF) instability and osteoarthritis, with high‐grade variants (Dejour types B‐D) conferring a particularly elevated risk of recurrent patellar dislocation and altered joint biomechanics.^1^, ^2^, ^3^, ^4^ The Dejour classification system, based on radiographic and magnetic resonance imaging (MRI) findings, remains the standard for categorizing TD severity and guiding treatment decisions in clinical practice.^1^, ^3^

Epidemiological and imaging studies consistently show a higher incidence of PF instability in women, accompanied by distinct differences in trochlear morphology.^2^, ^5^, ^6^, ^7^ Women with PF instability exhibit a shallower trochlear groove, increased sulcus angle, and greater trochlear asymmetry compared with men, even after normalization for knee size and skeletal maturity.^2^, ^7^ These differences are evident early in development and persist into adulthood, suggesting intrinsic sex‐based predisposition rather than acquired changes alone.^7^ Volumetric and 3‐dimensional analyses further confirm that the proximal trochlea is preferentially involved, and female patients show reduced trochlear volume and curvature, which may exacerbate instability.^6^, ^8^

Recent studies have also identified sex‐related variation in commonly used measurements of trochlear morphology.^5^, ^9^ In addition to trochlear morphology, other anatomical risk factors such as patella alta, increased tibial tubercle‐trochlear groove (TTTG) distance, and rotational malalignment frequently coexist and may further increase the risk of instability.^3^, ^4^, ^5^

Despite these insights, it remains unclear whether previously reported sex‐related anatomical differences are also present within a homogeneous cohort of patients with PF instability and high‐grade TD. Restricting the analysis to Dejour types B to D allows sex‐related differences to be evaluated within a population with comparable high‐grade trochlear morphology and avoids the heterogeneity introduced by including normal or low‐grade trochlear anatomy.

The purpose of this study was to assess sex‐related differences in PF anatomy among patients with PF instability and high‐grade TD (Dejour types B‐D). It was hypothesized that female patients with high‐grade TD would reveal more pronounced anatomical risk factors for PF instability when compared with their male counterparts.

## METHODS

Ethical approval was granted by the local research ethics committee, and all included patients gave their written consent.

### Patient Selection

Consecutive patients with symptomatic PF instability and high‐grade TD who were scheduled for surgical treatment between January 2010 and December 2020 at a single tertiary referral center were retrospectively identified. Eligibility required the availability of a preoperative lateral radiograph as well as an MRI of the affected knee. The diagnosis of TD was confirmed jointly by a musculoskeletal radiologist and an orthopaedic surgeon (J.A.) based on these imaging studies. Patients with normal trochlear morphology, type A dysplasia, missing preoperative MRI, or history of prior osseous procedures were excluded to minimize anatomical heterogeneity and to maintain the study focus on sex‐specific morphological differences in patients with high‐grade TD. PF instability was defined as recurrent lateral patellar dislocation after failure of nonoperative treatment based on clinical history, physical examination, and imaging findings. All indications for patellar stabilization surgery were established by the senior author (S.F.F.), Chief of Knee Surgery at Balgrist University Hospital and an experienced specialist in PF surgery. All procedures were either performed or directly supervised by the senior author. Surgical treatment was selected according to the individual anatomical abnormalities and established institutional treatment algorithms and consisted of medial PF ligament reconstruction alone or combined with tibial tubercle osteotomy, trochleoplasty, or both.

### Clinical and Radiographic Assessment

Patient demographics, including age at surgery, sex, body height, and body mass index, were extracted from clinical reports. In addition to demographic characteristics and TD classification, two independent reviewers (M.H., B.C.) assessed multiple radiographic and MRI‐based parameters, including tibiofemoral (TF) rotation, TTTG distance, sagittal TTTG (sTTTG) distance, tibial tubercle‐posterior cruciate ligament (TT‐PCL) distance, supratrochlear spur height, trochlear sulcus depth, Merchant's congruence angle, trochlear sulcus angle, patellotrochlear index, patellar tilt, patellar angle, patellar thickness, patellar width, and Caton‐Deschamps index (CDI).

TF rotation was measured on axial MRI as the angle between the tangent of the posterior femoral condyles and the tangent of the posterior tibial plateau.^10^ TTTG distance was defined as the perpendicular distance between the anterior‐most point of the tibial tuberosity and the deepest point of the trochlear groove, referenced to the tangent of the posterior condyles.^11^ The sTTTG distance was determined as the sagittal distance between the trochlear cartilage nadir and the anterior‐most point of the tibial tubercle at the patellar tendon insertion, oriented to the posterior condylar axis. An increased sTTTG value indicates that the tibial tubercle is positioned more posteriorly relative to the trochlear groove.^12^ The TT‐PCL distance was measured mediolaterally between the tibial tubercle midpoint and the medial border of the posterior cruciate ligament, parallel to the dorsal tibial condylar line.^13^ Unlike the TTTG distance, the TT‐PCL distance is less influenced by trochlear morphology and provides an alternative assessment of lateralization of the tibial tubercle.^13^ Supratrochlear spur height was defined as the maximal distance between a line along the anterior cortex of the distal femur and a parallel line through the tip of the supratrochlear spur. This parameter reflects the prominence of the supratrochlear bump and is a characteristic feature of TD.^14^

Trochlear sulcus depth was calculated as the perpendicular distance from the deepest trochlear point to a line connecting the highest points of the medial and lateral femoral condyles.^15^ Merchant's congruence angle was defined between a line through the trochlear groove nadir and a line connecting the anterior‐most points of the condyles.^16^ The trochlear sulcus angle was measured as the angle between two lines from the groove nadir to the highest points of the medial and lateral condyles on axial view.^1^

The patellotrochlear index was assessed on sagittal MRI and calculated as the percentage ratio of patellar articular cartilage overlapping the trochlear cartilage to the total patellar cartilage length.^17^ Patellar tilt was defined as the angle between the transverse axis of the patella and a line parallel to the posterior femoral condyles.^18^ The patellar angle was measured between the lateral patellar facet and the posterior condylar axis.^18^ Patellar thickness (bone + cartilage) was measured at its maximum point, typically in the axial midsection, whereas patellar width was defined at its widest dimension.^19^ CDI was calculated as the ratio of patellar tendon length (distance from the inferior patellar pole to the anterosuperior tibial plateau) to the length of the patellar articular surface.^20^

All measurements were independently performed by two observers (M.H., B.C.) using a picture archiving and communication system workstation certified for clinical use (MERLIN 7.1.22, Phönix‐PACS GmbH). In the event of disagreement, a third observer (J.A.) assessed the images to achieve consensus.

### Statistical Analysis

Sociodemographic and clinical characteristics of the study population were analyzed using descriptive statistics. Continuous variables are expressed as mean ± standard deviation. The normality of data distribution was assessed using the Shapiro‐Wilk test. Depending on the distribution characteristics, appropriate parametric or nonparametric tests were applied to evaluate differences in anatomical parameters between sexes. To account for within‐patient correlation arising from bilateral observations, multivariable linear mixed‐effects models were fitted with patient as a random intercept. Sex was included as the independent variable of interest. For patellar width, patellar thickness, and TT‐PCL distance, models were adjusted for age and body height. For patellar tilt and sTTTG distance, additional models were adjusted for age, body height, and established PF parameters potentially influencing these measurements, including supratrochlear spur height, CDI, sulcus depth, and TF rotation. Regression coefficients (*B*) are reported with 95% confidence intervals (CIs) and represent the adjusted mean difference between female and male patients. All statistical analyses were conducted using SPSS for Mac (Version 23.0, SPSS, Chicago, IL), with statistical significance defined as *P* < .05.

## RESULTS

The final cohort comprised 301 knees from 278 patients. Of these, 192 knees (63.8%) were from female patients. The mean age was 22.6 ± 8.1 years (range, 11‐50 years), and the mean body mass index was 24.9 ± 5.5 (range, 16.4‐48.1). Distribution according to the Dejour classification was 40.5% type B (n = 122), 31.2% type C (n = 94), and 28.2% type D (n = 85).

The anatomical parameters showing significant sex‐related differences are summarized in Table 1. Female patients had significantly smaller patellar width and thickness, greater patellar tilt, and increased sTTTG distance than male patients. TT‐PCL distance was also smaller in female patients but did not remain significant after adjustment. No significant sex‐related differences were observed for the remaining demographic and morphological parameters (Table 2).

**TABLE 1:** Anatomical Parameters Showing Significant Sex‐Related Differences

	**Men (n = 109)**	**Women (n = 192)**	***P*** **Value**
Patellar width, mm	41.3 ± 4.1	37.9 ± 3.4	<.001
Patellar thickness, mm	18.5 ± 2.6	16.8 ± 2.0	<.001
Patellar tilt, °	25.0 ± 10.8	29.3 ± 10.7	.001
TT‐PCL, mm	20.7 ± 4.0	19.2 ± 3.5	.001
sTTTG, mm	8.4 ± 6.0	9.9 ± 5.2	.005

*Note*: Values are presented as mean ± standard deviation. TT‐PCL was significant in the unadjusted analysis but not after adjustment for age and body height.

sTTTG, sagittal tibial tubercle‐trochlear groove; TT‐PCL, tibial tubercle‐posterior cruciate ligament.

**TABLE 2:** Demographic and Morphological Parameters Without Significant Sex‐Related Differences

	**Men (n = 109)**	**Women (n = 192)**	***P*** **Value**
Dislocation, yes	104 (95.4%)	171 (89.0%)	.085
Wiberg type			
1	17 (15.6)	40 (20.8)	.54
2	72 (66.1)	119 (62)	
3	20 (18.3)	33 (17.2)	
Dejour type			
B	45 (41.3)	77 (40.1)	.86
C	32 (29.4)	62 (32.3)	
D	32 (29.4)	53 (27.6)	
Age, yr	22.0 ± 6.7	23.0 ± 8.8	.93
BMI	25.2 ± 5.4	24.7 ± 5.5	.36
Patellar angle, °	124.2 ± 13.3	125.2 ± 12.7	.55
CDI	1.2 ± 0.2	1.2 ± 0.2	.71
PTI	41.8 ± 17.1	40.7 ± 14.7	.74
Sulcus angle, °	162.5 ± 7.0	161.9 ± 7.4	.51
Merchant's angle, °	38.3 ± 30.8	40.9 ± 26.8	.44
Sulcus depth, mm	2.0 ± 0.9	1.9 ± 0.9	.20
Spur height, mm	5.5 ± 1.8	5.2 ± 1.5	.20
TTTG, mm	15.3 ± 5.8	14.4 ± 4.4	.34
TF rotation, °	5.0 ± 7.9	5.3 ± 7.0	.73

*Note*: Values are presented as mean ± standard deviation or n (%). *P* < .05 was considered statistically significant.

BMI, body mass index; CDI, Caton‐Deschamps index; PTI, patellotrochlear index; TF, tibiofemoral; TTTG, tibial tubercle‐trochlear groove; yr, years

After adjustment for age and body height, female sex remained associated with smaller patellar width (*B* = −1.58 mm; 95% CI, −2.7 to −0.5; *P* = .004) and patellar thickness (*B* = −1.02 mm; 95% CI, −1.7 to −0.3; *P* = .004), as well as greater patellar tilt (*B* = 3.50°; 95% CI, 0.06‐7.0; *P* = .046) and sTTTG distance (*B* = 2.57 mm; 95% CI, 0.8‐4.4; *P* = .005). The association between female sex and TT‐PCL distance was no longer statistically significant after adjustment for age and body height (*B* = −1.01 mm; 95% CI, −2.2 to 0.2; *P* = .086). After additional adjustment for supratrochlear spur height, CDI, sulcus depth, and TF rotation, patellar tilt (*B* = 3.51°; 95% CI, 0.5‐6.5; *P* = .021) and sTTTG distance (*B* = 2.36 mm; 95% CI, 0.8‐4.0; *P* = .004) remained significantly greater in female patients.

## DISCUSSION

The principal finding of this study was that female patients with PF instability and high‐grade TD showed greater patellar tilt and sTTTG distance independent of age, body height, and relevant anatomical covariates. In addition, female patients exhibited smaller patellar width and thickness, whereas no adjusted sex‐related difference was observed for TT‐PCL distance. Previous studies have described sex‐related variation in trochlear morphology and other anatomical parameters associated with PF instability. The present analysis shows that such differences are also present within a cohort restricted to high‐grade TD. In particular, the associations of female sex with greater patellar tilt and sTTTG distance persisted after adjustment for the measured trochlear and rotational covariates.

Previous studies have consistently reported that women are more frequently affected by high‐grade TD and show sex‐specific thresholds for radiographic parameters such as sulcus angle and trochlear depth.^1^, ^5^, ^21^ The present study extends these observations by showing that female patients with high‐grade TD also exhibit greater patellar tilt and increased sTTTG distance. However, the present study assessed imaging morphology only and did not evaluate pain, cartilage status, or functional outcomes. Therefore, the observed anatomical differences should not be interpreted as explanations for sex‐related differences in symptoms or clinical outcomes. In addition, abnormal patellar tilt and increased sTTTG, reflecting a more posterior position of the tibial tuberosity, have been associated with higher PF contact pressures and early osteoarthritic changes.^12^, ^22^, ^23^, ^24^, ^25^ Whether these associations translate into clinically relevant sex‐specific differences cannot be determined from the present study.

The observed differences in patellar tilt and sTTTG persisted after adjustment for supratrochlear spur height, CDI, sulcus depth, and TF rotation. Thus, within this cohort, these associations were not explained by the measured features of TD and rotational alignment. Previous studies have similarly reported a more posterior tibial tubercle position in female patients, particularly in the presence of TD.^26^ The mechanism underlying this association remains uncertain. One possible explanation is that a more posterior position of the tibial tubercle relative to the trochlear groove may alter the line of action of the extensor mechanism, thereby influencing PF tracking and joint loading. However, this hypothesis cannot be confirmed by the present study because neither dynamic PF kinematics nor contact pressures were evaluated. Consistent with the present findings, a recent study reported greater sTTTG values in patients with high‐grade TD than controls, suggesting that altered sagittal tibial tubercle position may represent a characteristic feature of severe TD.^26^

The clinical implications of these findings should be interpreted with caution. Although previous studies have associated increased patellar tilt and higher sTTTG with PF maltracking and altered joint biomechanics,^1^, ^2^, ^3^, ^4^, ^5^, ^9^, ^15^, ^18^, ^27^, ^28^ the present study did not evaluate contact pressures, kinematics, or clinical outcomes. Therefore, it cannot be determined whether the observed anatomical differences are clinically meaningful or should influence treatment. Surgical decision‐making should continue to be based on each patient's individual anatomy rather than sex alone.

### Limitations

Several limitations must be considered when interpreting these findings. First, the retrospective study design and potential selection bias may have influenced the results. In addition, complete screening data, including the number of excluded patients and reasons for exclusion, were not available, precluding a comprehensive assessment of potential selection and transfer bias. Second, patient‐reported outcome measures were not included, which limits the ability to correlate sex‐specific morphological findings with clinical outcomes. Third, the models were adjusted for body height but not for direct measures of knee size, which may influence sex‐related differences in patellar dimensions. Fourth, subgroup analyses according to Dejour type may have been underpowered and susceptible to type II error. Fifth, the study was based on static imaging and did not assess dynamic PF kinematics, soft‐tissue laxity, or joint contact pressures. Finally, the findings may not be generalizable to nonsurgical patients, individuals with normal trochlear morphology, or patients with low‐grade TD.

## CONCLUSIONS

Among patients with PF instability and high‐grade TD, female sex was associated with greater patellar tilt and sTTTG distance after adjustment for age, body height, and relevant anatomical covariates.

## DECLARATION OF GENERATIVE AI AND AI‐ASSISTED TECHNOLOGIES IN THE WRITING PROCESS

During preparation of this article, the authors used ChatGPT and OpenEvidence to assist with language editing, translation, and literature searches. The authors subsequently reviewed and verified all content, references, and revisions and take full responsibility for the final article.

## DISCLOSURES

The authors (M.H., B.C., J.A., S.F.F.) declare that they have no known competing financial interests or personal relationships that could have appeared to influence the work reported in this article.

## References

[1] Tanaka MJ , Sodhi A , Wadhavkar I , et al. Redefining trochlear dysplasia: Normal thresholds vary by measurement technique, landmarks, and sex. Am J Sports Med. 2023;51:1202‐1210. 10.1177/03635465231158099 36942723

[2] Schroedter R , Koutp A , Guggenberger B , Svehlik M , Tschauner S , Kraus T . Anatomical risk patterns for patellofemoral instability in skeletally immature patients: A sex‐stratified MRI study. J Clin Med. 2025;14:5519. 10.3390/jcm14155519 PMC12347535 40807138

[3] Danielsen O , Poulsen TA , Eysturoy NH , Mortensen ES , Hölmich P , Barfod KW . Trochlea dysplasia, increased TT‐TG distance and patella alta are risk factors for developing first‐time and recurrent patella dislocation: A systematic review. Knee Surg Sports Traumatol Arthrosc. 2023;31:3806‐3846. 10.1007/s00167-022-07255-1 36854995

[4] Only AJ , Arendt EA , Hinckel BB . Anatomic risk factors for lateral patellar instability. Arthroscopy. 2024;40:2642‐2644. 10.1016/j.arthro.2024.08.009 39477653

[5] Balcarek P , Jung K , Ammon J , et al. Anatomy of lateral patellar instability: Trochlear dysplasia and tibial tubercle‐trochlear groove distance is more pronounced in women who dislocate the patella. Am J Sports Med. 2010;38:2320‐2327. 10.1177/0363546510373887 20713643

[6] Hodel S , Torrez C , Hoch A , Fürnstahl P , Vlachopoulos L , Fucentese SF . Increased femoral curvature and trochlea flexion in high‐grade patellofemoral dysplastic knees. Knee Surg Sports Traumatol Arthrosc. 2023;31:1361‐1369. 10.1007/s00167-022-07080-6 35920842

[7] Pruneski J , O’Mara L , Perrone GS , Kiapour AM . Changes in anatomic risk factors for patellar instability during skeletal growth and maturation. Am J Sports Med. 2022;50:2424‐2432. 10.1177/03635465221102917 35763558

[8] Voss A , Shin SR , Murakami AM , et al. Objective quantification of trochlear dysplasia: Assessment of the difference in morphology between control and chronic patellofemoral instability patients. Knee. 2017;24:1247‐1255. 10.1016/j.knee.2017.05.024 28666647

[9] DeVries CA , Bomar JD , Pennock AT . Prevalence of trochlear dysplasia and associations with patellofemoral pain and instability in a skeletally mature population. J Bone Joint Surg Am. 2021;103:2126‐2132. 10.2106/JBJS.20.01624 34546985

[10] Tensho K , Akaoka Y , Shimodaira H , et al. What components comprise the measurement of the tibial tuberosity‐trochlear groove distance in a patellar dislocation population? J Bone Joint Surg Am. 2015;97:1441‐1448. 10.2106/JBJS.N.01313 PMC7535107 26333740

[11] Schoettle PB , Zanetti M , Seifert B , Pfirrmann CW , Fucentese SF , Romero J . The tibial tuberosity‐trochlear groove distance: A comparative study between CT and MRI scanning. Knee. 2006;13:26‐31. 10.1016/j.knee.2005.06.003 16023858

[12] Ackermann J , Bergheim N , Hartmann M , Vlachopoulos L , Fucentese SF . Trochlear dysplasia is associated with increased sagittal tibial tubercle trochlear‐groove distance in patients with patellar instability. Arthroscopy. 2025;41:1002‐1008. 10.1016/j.arthro.2024.05.023 38844015

[13] Seitlinger G , Scheurecker G , Högler R , Labey L , Innocenti B , Hofmann S . Tibial tubercle‐posterior cruciate ligament distance: A new measurement to define the position of the tibial tubercle in patients with patellar dislocation. Am J Sports Med. 2012;40:1119‐1125. 10.1177/0363546512438762 22415209

[14] Giovannetti de Sanctis E , Mesnard G , Dejour DH . Trochlear dysplasia: When and how to correct. Clin Sports Med. 2022;41:77‐88. 10.1016/j.csm.2021.09.001 34782077

[15] Nacey NC , Fox MG , Luce BN , Boatman DM , Diduch DR . Assessing femoral trochlear morphologic features on cross‐sectional imaging before trochleoplasty: Dejour classification versus quantitative measurement. AJR Am J Roentgenol. 2020;215:458‐464. 10.2214/AJR.19.22400 32507014

[16] Fabricant PD , Heath MR , Mintz DN , et al. Many radiographic and magnetic resonance imaging assessments for surgical decision making in pediatric patellofemoral instability patients demonstrate poor interrater reliability. Arthroscopy. 2022;38:2702‐2713. 10.1016/j.arthro.2022.03.033 35398485

[17] Biedert RM , Albrecht S . The patellotrochlear index: A new index for assessing patellar height. Knee Surg Sports Traumatol Arthrosc. 2006;14:707‐712. 10.1007/s00167-005-0015-4 16496126

[18] Gobbi RG , Cavalheiro CM , Giglio PN , Hinckel BB , Camanho GL . Patellar tilt and patellar tendon‐trochlear groove angle present the optimum magnetic resonance imaging diagnostic reliability for patients with patellar instability. Arthroscopy. 2023;39:2339‐2351. 10.1016/j.arthro.2023.04.005 37116551

[19] Sullivan NP , Robinson PW , Ansari A , et al. Bristol index of patellar width to thickness (BIPWiT): A reproducible measure of patellar thickness from adult MRI. Knee. 2014;21:1058‐1062. 10.1016/j.knee.2014.07.007 25108841

[20] Huddleston HP , Redondo ML , Cregar WM , Christian DR , Hannon CP , Yanke AB . The effect of aberrant rotation on radiographic patellar height measurement using Canton‐Deschamps index: A cadaveric analysis. J Knee Surg. 2023;36:254‐260. 10.1055/s-0041-1731720 34261156

[21] Peeters W , Van Haver A , Van den Wyngaert S , Verdonk P . A landmark‐based 3D analysis reveals a narrower tibial plateau and patella in trochlear dysplastic knees. Knee Surg Sports Traumatol Arthrosc. 2020;28:2224‐2232. 10.1007/s00167-019-05802-x 31792598

[22] Ambra LF , Hinckel BB , Arendt EA , Farr J , Gomoll AH . Anatomic risk factors for focal cartilage lesions in the patella and trochlea: A case‐control study. Am J Sports Med. 2019;47:2444‐2453. 10.1177/0363546519859320 31287712

[23] Carlson VR , Boden BP , Sheehan FT . Patellofemoral kinematics and tibial tuberosity‐trochlear groove distances in female adolescents with patellofemoral pain. Am J Sports Med. 2017;45:1102‐1109. 10.1177/0363546516679139 PMC6006511 28029800

[24] Hanna FS , Teichtahl AJ , Wluka AE , et al. Women have increased rates of cartilage loss and progression of cartilage defects at the knee than men: A gender study of adults without clinical knee osteoarthritis. Menopause. 2009;16:666‐670. 10.1097/gme.0b013e318198e30e 19598333

[25] Kaiser P , Loth F , Attal R , et al. Static patella tilt and axial engagement in knee extension are mainly influenced by knee torsion, the tibial tubercle‐trochlear groove distance (TTTG), and trochlear dysplasia but not by femoral or tibial torsion. Knee Surg Sports Traumatol Arthrosc. 2020;28:952‐959. 10.1007/s00167-019-05588-y 31267191

[26] Bueno BT , Moore MR , Bi AS , et al. High‐grade trochlear dysplasia is associated with a more negative sagittal tibial tuberosity‐trochlear groove distance: A retrospective cohort study. Knee. 2025;57:1‐11. 10.1016/j.knee.2025.07.005 40782563

[27] Thouvenin C , Erard J , Abu Mukh A , Joseph L , Lustig S , Servien E . Patellar morphology is different in patellofemoral instability: An MRI comparative case‐control study. Knee. 2024;51:199‐205. 10.1016/j.knee.2024.09.007 39368299

[28] Abelleyra Lastoria DA , Hutchinson K , Tapadar T , et al. Patho‐morphology of patellar instability in children and adolescents: A systematic review and meta‐analysis. Knee. 2024;48:166‐196. 10.1016/j.knee.2024.03.009 38657526

